# *nifH* Gene Sequencing Reveals the Effects of Successive Monoculture on the Soil Diazotrophic Microbial Community in *Casuarina equisetifolia* Plantations

**DOI:** 10.3389/fpls.2020.578812

**Published:** 2021-01-25

**Authors:** Liuting Zhou, Jianjuan Li, Ganga Raj Pokhrel, Jun Chen, Yanlin Zhao, Ying Bai, Chen Zhang, Wenxiong Lin, Zeyan Wu, Chengzhen Wu

**Affiliations:** ^1^Fujian Agriculture and Forestry University, Fuzhou, China; ^2^College of Forestry, Fujian Agriculture and Forestry University, Fuzhou, China; ^3^Department of Chemistry, Birendra Multiple Campus, Tribhuvan University, Chitwan, Nepal; ^4^Key Laboratory of Crop Ecology and Molecular Physiology, Fujian Agriculture and Forestry University, Fuzhou, China; ^5^Fujian Provincial Key Laboratory of Agroecological Processing and Safety Monitoring, School of Life Sciences, Fujian Agriculture and Forestry University, Fuzhou, China

**Keywords:** *Casuarina equisetifolia*, monoculture rotations, *nifH* gene sequencing, diazotrophic microbial community, soil

## Abstract

The growth and productivity of *Casuarina equisetifolia* is negatively impacted by planting sickness under long-term monoculture regimes. In this study, Illumina MiSeq sequencing targeting *nifH* genes was used to assess variations in the rhizospheric soil diazotrophic community under long-term monoculture rotations. Principal component analysis and unweighted pair-group method with arithmetic means (UPGMA) clustering demonstrated distinct differences in diazotrophic community structure between uncultivated soil (CK), the first rotation plantation (FCP), the second rotation plantation (SCP), and the third rotation plantation (TCP). Taxonomic analysis showed that the phyla *Proteobacteria* increased while *Verrucomicrobia* decreased under the consecutive monoculture (SCP and TCP). The relative abundance of *Paraburkholderia*, *Rhodopseudomonas*, *Bradyrhizobium*, *Geobacter*, *Pseudodesulfovibrio*, and *Frankia* increased significantly while *Burkholderia*, *Rubrivivax*, and *Chlorobaculum* declined significantly at the genus level under consecutive monoculture (SCP and TCP). Redundancy analysis (RDA) showed that *Burkholderia, Rubrivivax*, and *Chlorobaculum* were positively correlated with total nitrogen and available nitrogen. In conclusion, continuous *C. equisetifolia* monoculture could change the structure of diazotrophic microbes in the rhizosphere, resulting in the imbalance of the diazotrophic bacteria population, which might be a crucial factor related to replanting disease in this cultivated tree species.

## Introduction

*Casuarina equisetifolia* Forst has covers over 300,000 ha in southeastern China. It is one of the preferred trees for forming shelterbelts in coastal areas, as it vegetates the coasts and contributes to stabilizing coastal sand and protecting against storms (Zhong et al., 2005; Karthikeyan et al., 2013). However, continuous monocultures of *C. equisetifolia* result in productivity decline and regeneration failure. This phenomenon is referred to as the consecutive monoculture problem (CMP) (Wardle et al., 2004). Replanting diseases are observed in many cultivated tree species, including Chinese fir, *Pinus elliottii*, *Picea abies*, and *Eucalyptus* spp. (Wu et al., 2017). The depletion in soil nutrients (Bennett et al., 2012), the autointoxication of root exudations (Araya et al., 2012), and the imbalance of rhizospheric microflora (Wu et al., 2016a) are thought to be the main reasons for CMP. Increasing the amount of chemical fertilizer does not alleviate this problem (Wardle et al., 2004). Root exudates not only directly inhibit the growth and reproduction of plants but also restrain rhizosphere microorganisms (Li et al., 2014; Arafat et al., 2017). Great attention has been paid to rhizospheric biological processes in recent years. Lu et al. (2017) indicated that soil microbial community compositions and theirs structures had distinct variations under Poplar monocultures. Similar results were found in Chinese fir, *Eucalyptus*, and *Pinus halepensis* (Fernandez et al., 2008; Jie et al., 2014; Wu et al., 2017).

Biological nitrogen fixation has attracted widespread attention due to its influential role in the nitrogen cycle. Hsu and Buckley (2009) investigated the diversity of nitrogen-fixing genes and their functional microbial communities through high-throughput MiSeq amplicon sequencing of *nifH* gene, which encodes a subunit of the nitrogenase enzyme complex related to nitrogen fixation efficiency. Penton et al. (2016) amplified *nifH* gene sequencing and found that permafrost thawing in Alaskan soil altered the N-fixing microbial community composition in soil as the depth of groundwater changed. Wang et al. (2017) found that long-term inorganic fertilization in acid soil altered the diazotrophic community structure and decreased the abundance of operational taxonomic units (OTUs). Biological nitrogen fixation has been recognized as a crucial source of nitrogen to support certain primary production (Zehr et al., 1998). N-fixation by diazotrophs is an important strategy by which most organisms regulate biological productivity (Li et al., 2018). Nevertheless, the relationship between the diazotrophic microbial community and monocultures of *C. equisetifolia* has received little attention. On the basis of the above facts, we hypothesized that diazotrophic microbial community structure might be altered by *C. equisetifolia* monocultures (Wu et al., 2016b; Chen et al., 2017; Zhou et al., 2019).

In this study, quantitative PCR (qPCR) assays and the MiSeq high-throughput sequencing technique were applied to assess the shifts in the abundance of *nifH* gene and diazotrophic community composition in rhizosphere soil, respectively, after *C. equisetifolia* monoculture. Meanwhile, correlation analyses were used to resolve the relationships between the diazotrophic microbial community and environmental parameters, including total nitrogen (TN), total phosphorus (TP), total potassium (TK), available nitrogen (AN), available phosphorus (AP), available potassium (AK), and pH. This research will help improve our understanding of the nitrogen-fixing microbial community structure in soil under CMP. It will provide effective scientific-based references on the molecular ecological mechanisms of soil restoration and improvement.

## Materials and Methods

### Research Plot and Sample Collection

In this study, the plot was located at Chihu National Forest Center in Fujian Province, China (24°54′N and 118°55′E). Since the 1960s, the Chihu Forestry Farm cultivated a large-scale coastal windbreak and gradually established a settled coastal shelterbelt system. The annual mean temperature is 19.8°C (the extreme high and low temperatures are 35°C and 1°C, respectively), with a mean annual precipitation of 1,029 mm and a mean annual evaporation of 2,000 mm. There are three generations of *C. equisetifolia* plantation in the forest farm that were planted in 1987 (first rotation plantation, FCP), 2011 (second rotation plantation, SCP), and 2014 (third rotation plantation, TCP). Due to the long growth period of the arbor and field condition, the method of “space replacing time” is often applied in forestry research (Shi et al., 2018; Deng et al., 2019).

Three sampling positions (20 m × 20 m) were established at the FCP, SCP, and TCP on January 6, 2019. At the same time, a vacant area of soil in the forest farm with no *C. equisetifolia* cultivation was selected as a blank control, or CK. Each sampling position was set with three duplicate quadrats for a total of 12 quadrats. Soil samples were randomly collected from a depth of 0–20 cm at 12 quadrats. Twenty random replicated samples were taken from each quadrat. Soil samples from the FCP, SCP, and TCP were collected from the rhizosphere of *C. equisetifolia*. The field samplings were performed according to the method described by Zhou et al. (2019).

### Determination of Soil Nutrients

Soil pH was measured using a glass electrode pH meter (1:2.5 soil–water suspensions) (Zhao et al., 2017). The available and total amounts of NPK were determined referring to the methods by Jackson (2005).

### DNA Extraction

Soil DNA extractions were completed using a Power Soil DNA Isolation Kit (MoBio Laboratories, Carlsbad, CA, United States) following the manufacturer’s specifications. The genomic DNA was confirmed using 1.2% agarose gels.

### qPCR for *nifH*

Quantitative PCR was performed to quantify the relative abundance of the *nifH* gene in four individual rhizospheric soils with the primers *nifH*-F (AAAGGYGGWATCGGYAART CCACCAC) and *nifH*-R (TTGTTSGCSGCRTACATSGCCAT CAT) (Zhang et al., 2016). The reaction mixture (15 μl) for qPCR consisted of 7.5 μl 2 × SYBR green I Super Real Premix (TIANGEN, Beijing, China), 10 μM of each primer and 40 ng template DNA. The amplification conditions were 95°C for 30 s, followed by 40 cycles of 95°C for 5 s, 60°C for 40 s. The standard curve equation was Y = −4.083X + 46.196 (R^2^ = 0.99).

### PCR Amplification and *nifH* Sequencing

High-throughput sequencing was used to explore the influences of successive monocultures of *C. equisetifolia* on rhizospheric soil diazotrophic microbial communities. For *nifH* gene sequencing, the primers *nifH*-F and *nifH*-R (the same as above) were applied to amplify the *nifH* gene. The PCR amplification was implemented on a Mastercycler Gradient (Eppendorf, Germany) with a 25 μl volume, including 12.5 μl 2 × Taq PCR MasterMix, 3 μl BSA (2 ng/μl), 2 μl Primer (5 μM), 2 μl template DNA, and 5.5 μl ddH_2_O. Cycling parameters were 95°C for 5 min, followed by 32 cycles of 95°C for 45 s, 55°C for 50 s, and 72°C for 45 s with a final extension at 72°C for 10 min. The amplicons were purified with a QIAquick Gel Extraction Kit (QIAGEN, Germany), sequenced on the MiSeq PE300 platform at Allwegene Company, Beijing, China.

### Data Analyses

The low-quality (*q* < 20) and short sequences (<200 bp) were removed *via* the Pear (v. 0.9.6). The final length of sequences was 400–420. The datasets were then analyzed using QIIME (v. 1.8.0, Caporaso et al., 2010). The qualified sequences were clustered into OTUs at a 97% identity threshold (Edgar, 2013). All effective tags were classified into different taxonomic groups using the Ribosomal Database Project (RDP) classifier (Cole et al., 2009). Alpha diversity indices were employed to identify the richness and diversity within samples (Xiong et al., 2015). In order to make the sequencing depth consistent, the abundances of OTUs were normalized. Alpha diversity indices were calculated by R software (v. 2.15.3, Caporaso et al., 2010) based on the normalized data (21,778 taxa). The principal component analysis (PCA), clustering analyses, and non-metric multidimensional scaling (NMDS) were carried out to assess the similarity and difference between individual samples (Wang et al., 2012). PCA and NMDS were implemented based on the R with vegan package (v. 3.0.2, Oksanen et al., 2009; Goossen et al., 2010). To compare the diazotrophic community compositions and structures in the rhizosphere, heat maps were applied within the top 20 most abundant OTUs using Mothur (Jami et al., 2013). Redundancy analysis (RDA) was performed to establish which environmental parameters played an important role in the variation of top 12 most abundant OTUs using Canoco 5.0. Statistical analysis was carried out by DPS 7.05 software, and analysis of variance (ANOVA) was applied to identify significance difference by LSD’s test (*P* < 0.05).

## Results

### Soil Physicochemical Data

The results of soil nutrient analysis showed that replanting soils had a lower level of pH under the extended *C. equisetifolia* monoculture regime. The TN content was higher in the FCP than in the SCP (significant, *P* < 0.05) and TCP (not significant, *P* > 0.05). AN was higher in the FCP than in the SCP and TCP (*P* > 0.05). In the FCP and SCP, TP was significantly higher than in the TCP (*P* < 0.05). No significant differences were observed in AP, TK, and AK (Supplementary Table 1).

### Abundance of the *nifH* Gene

**FIGURE 1:**
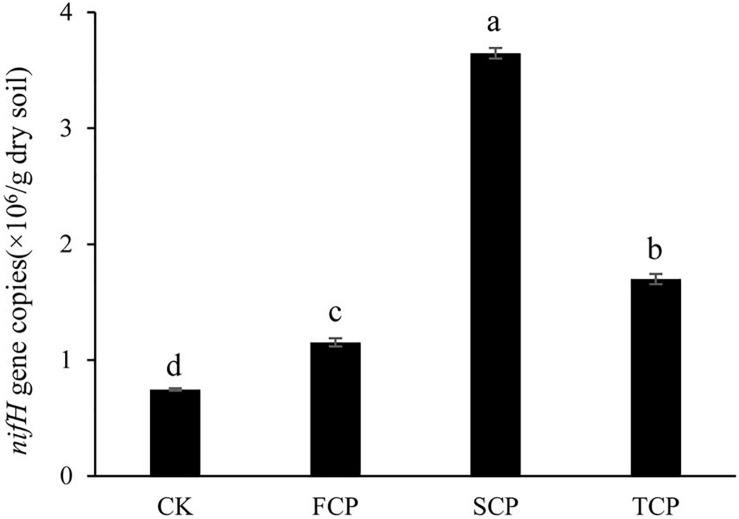
Quantification of *nifH* gene in four different soil samples. Vertical bars show standard deviations. CK, FCP, SCP, and TCP represent the control with no *C. equisetifolia* cultivation, the first rotation plantation, the second rotation plantation and the third rotation plantation.

Figure 1 indicates that the quantity of the *nifH* gene changed significantly under monoculture regime (*p* < 0.05). The number of copies of the *nifH* gene was between 0.746 × 10^6^ and 3.647 × 10^6/^g soil, and the highest and lowest numbers were found in the SCP and CK, respectively. Compared with the CK, the number of copies in the FCP, SCP, and TCP were increased by 54.4, 388.9, and 127.6%, respectively.

### OTU Clusters and Species Annotation

After filtration analysis, a sum of 656,599 high-quality tags (clean tags) was acquired from the CK, FCP, SCP, and TCP, with a mean of 54,717 effective sequences (Supplementary Table 2). Rarefaction curves indicated that the number of observed species was stable over 20,000 tags (Supplementary Figure 1). At the 97% similarity cut-off, effective tags from 12 soil samples were clustered into 4,610 OTUs. The average OTU numbers in the CK, FCP, SCP, and TCP were 272, 332, 375, and 559, respectively (Supplementary Table 2). The raw sequences data was deposited in NCBI (PRJNA666767).

### Alpha Diversity Indices

In this research, the richness and diversity of diazotrophic microbial communities were obtained according to 21,778 taxa. Table 1 exhibits the Chao1, Observed species, PD whole tree, and Shannon index of nitrogen-fixing microbial communities. The observed number of species was dramatically higher in the TCP than in the FCP and SCP (*p* < 0.05). The Shannon index was significantly higher in the TCP than in the FCP (*p* < 0.05) and it was also higher than in the SCP (not significant, *p* > 0.05). In the TCP, the soil nitrogen-fixing microbial communities showed significantly higher Chao1 and PD whole tree values than in the FCP and SCP (*p* < 0.05) (Table 1).

**TABLE 1:** Calculations of Alpha diversity indices in CK, FCP, SCP, and TCP.

	Chao1	Observed species	PD whole tree	Shannon
CK	347.122 ± 3.529d	268.533 ± 9.411c	96.189 ± 4.781a	4.711 ± 0.086ab
FCP	501.560 ± 12.173b	353.267 ± 28.163b	34.525 ± 3.036d	4.461 ± 0.075b
SCP	439.649 ± 9.178c	350.867 ± 6.438b	54.372 ± 1.389c	4.738 ± 0.098ab
TCP	657.198 ± 11.229a	558.000 ± 5.667a	66.642 ± 2.091b	5.192 ± 0.406a

*Different letters in each column indicate significant differences (*P* ≤ 0.05 and *n* = 3).*

### PCA, UPGMA Clustering, NMDS, and PERMANOVA

**FIGURE 2:**
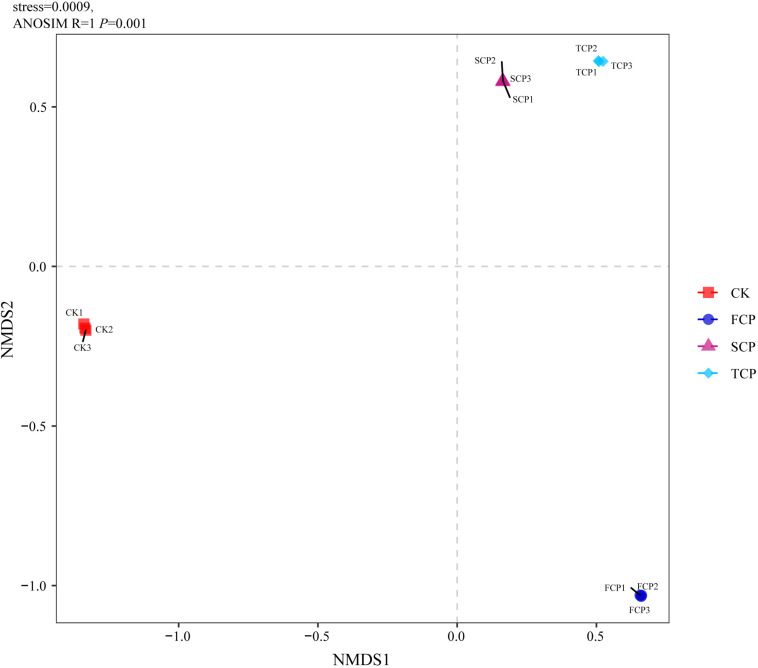
Non-metric multidimensional scaling (NMDS) ordination for CK, FCP, SCP, and TCP.

The PCA analysis indicated obvious dissimilarities in the soil diazotrophic community structures among the four soil samples. The PCA plot completely accounted for 72.56% of the overall variation in the soil nitrogen-fixing microbial community. The PC1 explained 47.85% and the PC2 explained 24.71% of the microbial variation (Supplementary Figure 2A). When observing the unweighted pair-group method with arithmetic means (UPGMA) clustering, the figure showed that diazotrophic community structures clustered together from the SCP and TCP, whereas they were separated from the FCP, and the CK formed a separate group (Supplementary Figure 2B). The results of PCA and UPGMA clustering revealed that diazotrophic microbial community composition is different under monoculture regimes. Non-metric multidimensional scaling (NMDS) was applied to illustrate the differences in diazotrophic community composition and structure (Tibbits, 2008). Pairwise contrasts demonstrated that the CK, FCP, SCP, and TCP plots were significantly (*P* < 0.05) separated (Figure 2). The PERMANOVA analysis showed significant differences in the composition and distribution of the diazotrophic microbial community (*F* = 26.7519 and *P* = 0.001).

### Venn Diagram Analysis

The shared and exclusive OTUs among the FCP, SCP, and TCP were explored through Venn analysis. Supplementary Figure 3 indicates that the proportion of OTUs shared in the FCP, SCP and TCP was 30.9% (323 species). The proportion of OTUs found only in the FCP was 5.4% (57 species). The abundance of OTUs exclusively constructed in the SCP accounted for up to 13.4% (140 species). The percentage of OTUs constructed only in the TCP was 23.8% (249 species).

### Abundance Change in the Diazotrophic Microbial Communities

Diazotrophic microbial OTUs consisted mostly of five phyla: *Proteobacteria*, *Verrucomicrobia*, *Chlorobi*, unidentified, and *Actinobacteria*. Among them, *Proteobacteria* was the dominant phylum, accounting for 85.7, 65.7, 94.4, and 85.5% of the total species in the CK, FCP, SCP, and TCP, respectively (Supplementary Figure 4).

At genus level, the *C. equisetifolia* monoculture regime generated a distinct enhancement in the relative abundance of *Paraburkholderia*, *Rhodopseudomonas*, *Bradyrhizobium*, *Geobacter*, *Pseudodesulfovibrio*, and *Frankia* and a distinct reduction in *Burkholderia*, *Rubrivivax*, and *Chlorobaculum* (Table 2). Compared with the FCP, the genus *Desulfovibrio* was slightly higher in the SCP but decreased in the TCP. The most common diazotrophic microbial communities of *C. equisetifolia* are listed in Table 2, including *Paraburkholderia*, *Bradyrhizobium*, *Burkholderia*, and *Frankia*.

**TABLE 2:** Calculations of the top diazotrophic bacteria for four different soil samples at the genus level.

	CK	FCP	SCP	TCP
*Paraburkholderia*	0.50 ± 0.33b	6.05 ± 1.36b	29.58 ± 0.83a	40.18 ± 14.09a
*Rhodopseudomonas*	32.75 ± 3.34a	0.25 ± 0.04c	5.75 ± 1.50b	1.69 ± 1.39bc
*Bradyrhizobium*	8.04 ± 3.26b	1.87 ± 0.41c	21.24 ± 2.32a	8.21 ± 1.75b
Unidentified	10.23 ± 2.88b	18.52 ± 2.01a	3.87 ± 0.76c	4.91 ± 0.74c
*Burkholderia*	0.34 ± 0.49b	20.51 ± 3.52a	2.84 ± 0.34b	3.77 ± 0.34b
*Geobacter*	0.81 ± 0.22c	3.46 ± 0.77bc	11.64 ± 2.36a	7.71 ± 2.32ab
*Rubrivivax*	0.11 ± 0.07b	17.86 ± 3.07a	2.31 ± 0.11b	2.94 ± 0.54b
*Halorhodospira*	16.07 ± 1.42a	0.31 ± 0.12b	2.27 ± 0.35b	0.96 ± 0.61b
*Desulfovibrio*	2.39 ± 1.03c	6.02 ± 1.01ab	6.56 ± 1.16a	3.22 ± 1.26bc
*Chlorobaculum*	0.71 ± 0.83b	15.07 ± 1.97a	0.82 ± 0.16b	0.61 ± 0.12b
*Pseudodesulfovibrio*	2.46 ± 0.98ab	0.97 ± 0.34b	3.46 ± 0.85ab	5.89 ± 2.29a
*Frankia*	0.15 ± 0.10b	0.38 ± 0.32b	0.34 ± 0.14b	8.70 ± 2.77a

*Different letters in each column indicate significant differences (*P* ≤ 0.05 and *n* = 3).*

**FIGURE 3:**
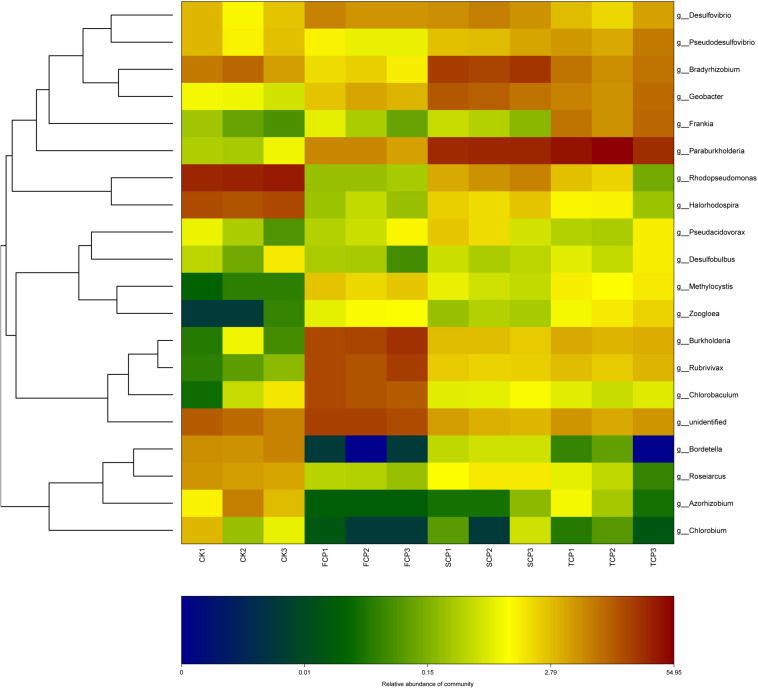
Heat map analysis of the dominant diazotrophic genera in four individual soil samples.

It can be seen from the heat map analysis of the top 20 diazotrophic genera that distinct variations in diazotrophic community structure arose with the increase of CMP. Compared with the FCP, the discrepancy of community structures of diazotrophic bacteria increased within the extended monoculture regime, implying that diazotrophic community structure gradually changed under the CMP (Figure 3).

**FIGURE 4:**
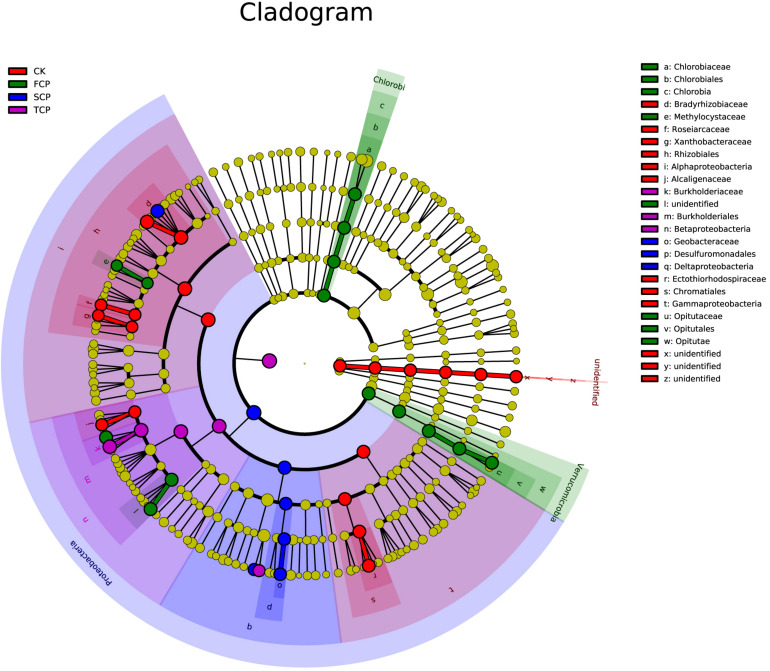
Phylogenetic dendrogram of biomarkers in C. equisetifolia rhizospheric soil diazotrophic bacteria in CK, FCP, SCP, and TCP. The circles from inside to outside exhibit diazotrophic taxonomic levels from phylum to genus. Yellow dots mean that there is no significant difference in diazotrophic abundance among individual soil samples. The colors of the biomarkers are based on their corresponding class colors on the right.

Linear discriminant analysis effect size (LEfSe) was applied to identify biomarkers (potential discriminating species in relative abundance between groups) from metagenome taxa (Xia et al., 2018). The LEfSe analysis revealed that there were 30 potential bacterial markers distinguishing the CK, FCP, SCP, and TCP with linear discriminant analysis (LDA) scores more than 3. In these tests, one phylum (unidentified), three classes (*Alphaproteobacteria*, *Gammaproteobacteria*, and unidentified), three orders (*Rhizobiales*, *Chromatiales*, and unidentified), and six families (*Bradyrhizobiaceae*, *Roseiarcaceae*, *Xanthobacteraceae*, *Alcaligenaceae*, *Ectothiorhodospiraceae*, and unidentified) were biomarkers for CK. Two phyla (*Chlorobi* and *Verrucomicrobia*), two classes (*Chlorobia* and *Opitutae*), two orders (*Chlorobiales* and *Opitutales*), and four families (*Chlorobiaceae*, *Methylocystaceae*, *Opitutaceae*, and unidentified) were significantly different in the FCP. In the SCP, biomarkers were mostly clustered in *Proteobacteria*, including *Deltaproteobacteria*, *Desulfuromonadales*, and *Geobacteraceae*. In the TCP, biomarkers were *Betaproteobacteria*, *Burkholderiales*, and *Burkholderiaceae* (Figure 4).

### Correlation Analysis Between Diazotrophic Microbial Communities and Environmental Parameters

**FIGURE 5:**
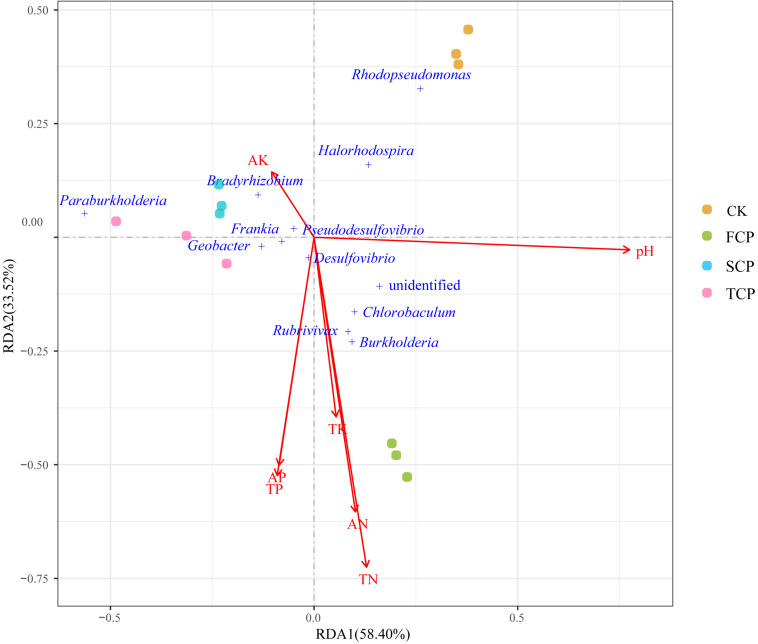
RDA of soil physicochemical properties and predominant nitrogen-fixing microbial communities in four individual soil samples.

To detect the relationships between diazotrophic microbial communities and environmental parameters, RDA was performed based on the top 12 species of the diazotrophic microbial community and environmental parameters (Figure 5). The influence of environmental factors on the diazotrophic microbial community can be seen from the length of the arrow and the angle between the arrow and the microbe. RDA canonical axes 1 and 2, respectively, described 58.40 and 33.52% of the variation in the diazotrophic microbial community, suggesting a remarkable correlation between microbial communities and environmental parameters. As shown in Figure 5, the SCP and TCP were clustered together and distributed at the negative end of RDA1, away from the CK and FCP, reflecting the significant shift of the diazotrophic microbial community under a monoculture regime. The results of RDA were consistent with the PCA results (Supplementary Figure 2B). In addition, from the results in Figure 5, it can be seen that *Burkholderia, Rubrivivax* and *Chlorobaculum* were positively correlated with the environmental parameters of TN, AN and TK, but negatively correlated with AK. However, the opposite was true for the *Bradyrhizobium* which was positively correlated with AK.

## Discussion

Consecutive monoculture problem, also known as soil sickness, has a severe impact on the growth and health of plants. It is a widespread and complex phenomenon in numerous cultivated tree species (Huang et al., 2013). Many factors are thought to contribute to the continuous cropping problem, including the physical and chemical properties of soil, the accumulation of allelochemicals, and changes in the soil microbial community (Wu et al., 2016a; Chen et al., 2018). In this study, we found that the contents of soil nitrogen (AN and TN) under monoculture regimes (SCP and TCP) were significantly lower than that in the FCP (Supplementary Table 1). The results indicated that soil physical and chemical properties may indirectly affect the healthy growth of *C. equisetifolia* through regulating the structure of the soil microbial community (Berendsen et al., 2012).

Recently, many studies have revealed that shifts in the soil microbial community are related to this soil sickness (Li et al., 2014; Liu et al., 2017). Soil microbes play a vital role in the growth and health of plants. Soil microbes are also known as the second genome of plants (Berendsen et al., 2012; Berg et al., 2017). N-fixing organisms play a profound part in nitrogen cycling in forest ecosystems (Yousuf et al., 2014). In the present study, the *nifH* gene abundance in *C. equisetifolia* increased with the extension of monoculture. These results are contradictory to those of a previous study on a *Eucalyptus* successive plantation (Monteiro et al., 2020). Species diversity is a crucial factor for soil health (Chaparro et al., 2012). Wu et al. (2017) reported that the rhizosphere microbial diversity in Chinese fir was decreased after long-term successive rotations. In this study, most of the diversity indices were significantly lower in the FCP and in the SCP than TCP (Table 1). The differing results among these studies may be due to differences in factors including plant types, soil environments, and root chambers. The number of copies of the *nifH* gene increased under monoculture regime, while diversity indices were increased or decreased with monoculture. That is the diazotrophic community compositions and structures were changed under long-term monoculture *C. equisetifolia* plantations, indicating that our hypothesis was correct.

In recent years, more attention has been paid to the relationship between key rhizosphere microbes and plants, which is central to plant growth and development (Mendes et al., 2011; Chen et al., 2017). The *Proteobacteria* phylum is commonly explored in soil systems (Gaby and Buckley, 2011). In this study, the most predominant phylum present in the rhizosphere was *Proteobacteria*. The diazotrophic bacterial populations in the *C. equisetifolia* rhizospheric soil (Supplementary Figure 4) accounted for 85.69, 65.75, 94.40, and 85.54% of the total in the CK, FCP, SCP, and TCP, respectively, which was in accordance with the results found by Bazylinski et al. (2013). Taxonomic analysis (genus level) showed that continuous monoculture of *C. equisetifolia* increased the relative abundance of *Paraburkholderia*, *Rhodopseudomonas*, *Bradyrhizobium*, *Geobacter*, *Pseudodesulfovibrio*, and *Frankia*, whereas the abundance of *Burkholderia*, *Rubrivivax*, and *Chlorobaculum* decreased significantly (Table 2). Among these diazotrophs, compared with the FCP, the relative abundance of *Bradyrhizobium* increased by 1,135.83 and 439.04% in the SCP and TCP, respectively. *Bradyrhizobium* was the dominant diazotroph in soil systems due to strong persistence of diazotrophic bacteria across varying soil environmental conditions (Pereira et al., 2013). Relative to the FCP, the abundance of *Burkholderia* decreased by 722.18 and 544.03% in the SCP and TCP, respectively. *Burkholderia* can be used for biological control through such methods as promoting plant growth and bio-remediation. It has been reported that *Burkholderia* has the ability of nodulation and N-fixation in certain legume plants including *Burkholderia vietnamiensis*, *Burkholderia phymatum*, and *Burkholderia tuberum* (Coenye and Vandamme, 2003). Wu et al. (2016a) reported that monoculture of *Pseudostellaria heterophylla* led to a significant decline in the abundance of *Burkholderia*. Mendes et al. (2011) showed that the relative abundances of *Burkholderiaceae* was positively associated with disease suppression of sugar beet. Thus, certain species of diazotrophic bacteria found in this study were inhibited under *C. equisetifolia* monocultures (SCP and TCP) that might be key rhizosphere microbes or plant growth promoters. This issue should be studied in the future.

Many reports have indicated that soil microbial community composition was indirectly influenced by environmental parameters (Li and Wu, 2018; Zhang et al., 2019). In our study, RDA results showed that key rhizosphere microbes (*Burkholderia, Rubrivivax* and *Chlorobaculum*) were positively correlated with soil nitrogen (AN and TN). Therefore, based on their abundance (a significant decrease in SCP and TCP), soil nitrogen seems to play an important role in *C. equisetifolia* monocultures. The decline of *Burkholderia, Rubrivivax* and *Chlorobaculum* may be related to soil nitrogen under continuous planting. Their abundance may indicate an important role in plant-microbial interactions and soil function as well as nitrogen fixation. In addition, *Burkholderia* is considered a rhizospheric-plant promoting group (Zhao K. et al., 2014). It could play an important role in maintaining the stability of soil communities (Wakelin et al., 2017). The results of RDA suggested that the diazotrophic microbial community may respond differently to environmental parameters, but this hypothesis should be further tested.

A growing number of researchers have reported that the imbalance of key rhizosphere microflora is considered to be the main cause of CMP. Zhao J. et al. (2014) revealed that *Eucalyptus* monocultures increased the abundance of fungi in rhizospheric soil. Successive rotations of *Cunninghamia lanceolata* generated imbalance in the soil microbial community. Most studies have reported that this phenomenon of imbalance resulted from alterations of the rhizospheric microflora induced by plant root exudations rather than direct allelopathy (Li et al., 2014; Wu et al., 2015). In this study, the continuous monoculture of *C. equisetifolia* shifted the structure of the diazotrophic population in rhizospheric soils. This shift may result in the imbalance of diazotrophic bacteria population structure, which might be a crucial factor in replanting disease of this cultivated tree species. Many previous studies have shown that root exudates restructure the plant-associated rhizospheric microbes and these microbes impact plants (Paterson et al., 2007; Haichar et al., 2008; Michalet et al., 2013). Therefore, further studies are required to analyze the effects of the *C. equisetifolia* root exudates to obtain precise knowledge of the soil nitrogen-fixing microorganism community structure.

## Conclusion

Our result indicates that the continuous monoculture of *C. equisetifolia* distinctly influenced the diazotrophic community compositions and structures in the rhizosphere. The phyla *Proteobacteria* increased, whereas *Verrucomicrobia* decreased with increasing continuous monoculture. At the genus level, the relative abundance of *Paraburkholderia*, *Rhodopseudomonas*, *Bradyrhizobium*, *Geobacter*, *Pseudodesulfovibrio*, and *Frankia* increased significantly, while *Burkholderia*, *Rubrivivax*, and *Chlorobaculum* declined significantly. To more efficiently explore the causes of obstacles to continuous monoculture, the isolation of specific N-fixing organisms (i.e., *Bradyrhizobium* and *Burkholderia*) and their functions, which may be correlated with CMP, should be further studied. It is noteworthy that most specific N-fixing organisms appear to be highly sensitive to changes in the soil under monoculture regimes. This should be investigated further.

## Data Availability Statement

The data presented in the study are deposited in the (NCBI) repository, accession number (PRJNA666767; SRP286063).

## Author Contributions

ZW, WL, and LZ conceived the study. ZW, YZ, LZ, and WL designed all the experiments. JL, JC, YB, and CZ performed the experiments. LZ, CW, GP, and JL performed the statistical analyses. LZ and ZW wrote the manuscript with the assistance and approval of all authors.

## Conflict of Interest

The authors declare that the research was conducted in the absence of any commercial or financial relationships that could be construed as a potential conflict of interest.
