# Supplementary material for: nifH Gene Sequencing Reveals the Effects of Successive Monoculture on the Soil Diazotrophic Microbial Community in Casuarina equisetifolia Plantations
Source: Front Plant Sci. 2021 Jan 25;11:578812. doi: 10.3389/fpls.2020.578812 (PMC7869410; doi:10.3389/fpls.2020.578812)
Supplement: Supplementary file 1 [file Data_Sheet_1.docx]

**
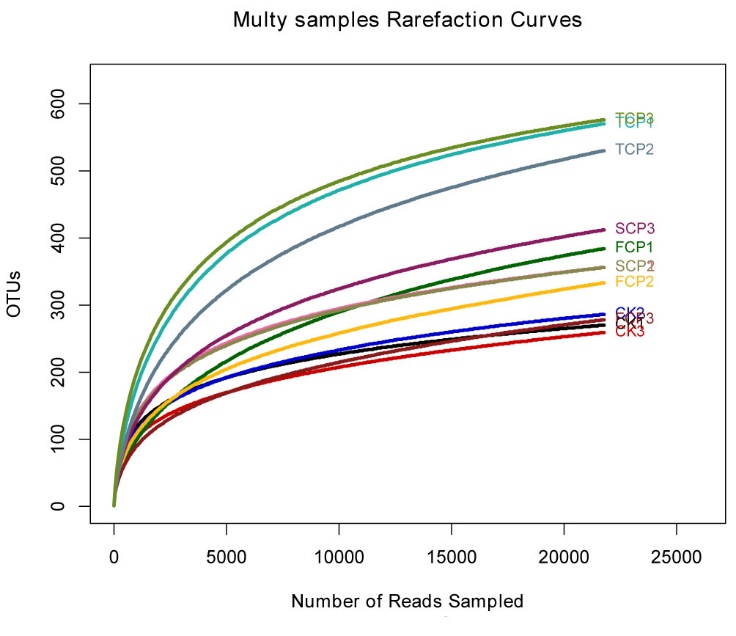
**

**Figure S1．**Rarefaction curves of nitrogen-fixing microbial communities according to the observed OTUs at 97% taxas similarity in different rhizospheric soil.


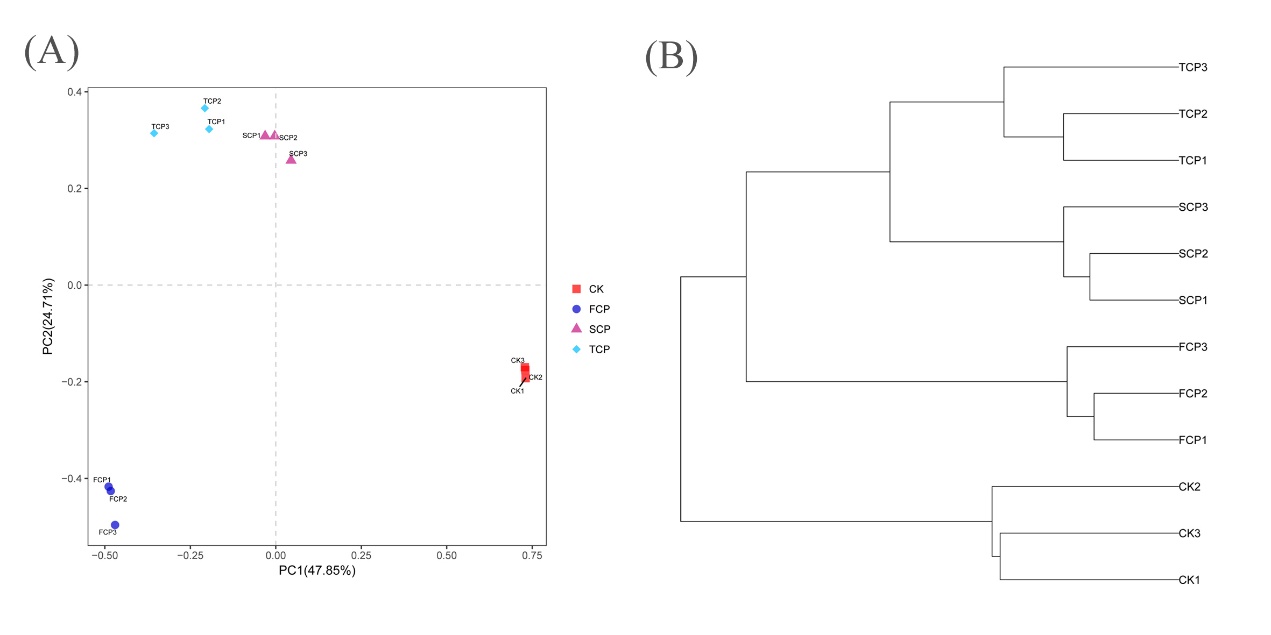


**Figure S2．**PCA analysis (a) and UPGMA clustering (b) of nitrogen-fixing microbial community for four individual rhizospheric soil samples.


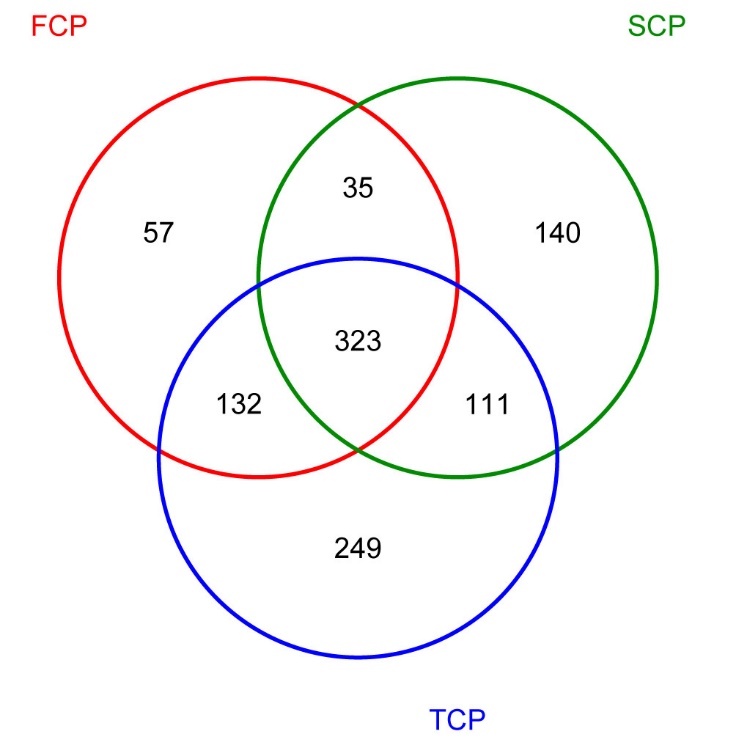


**Figure S3.** Venn analysis of shared and exclusive OTUs among four individual rhizospheric soil samples.


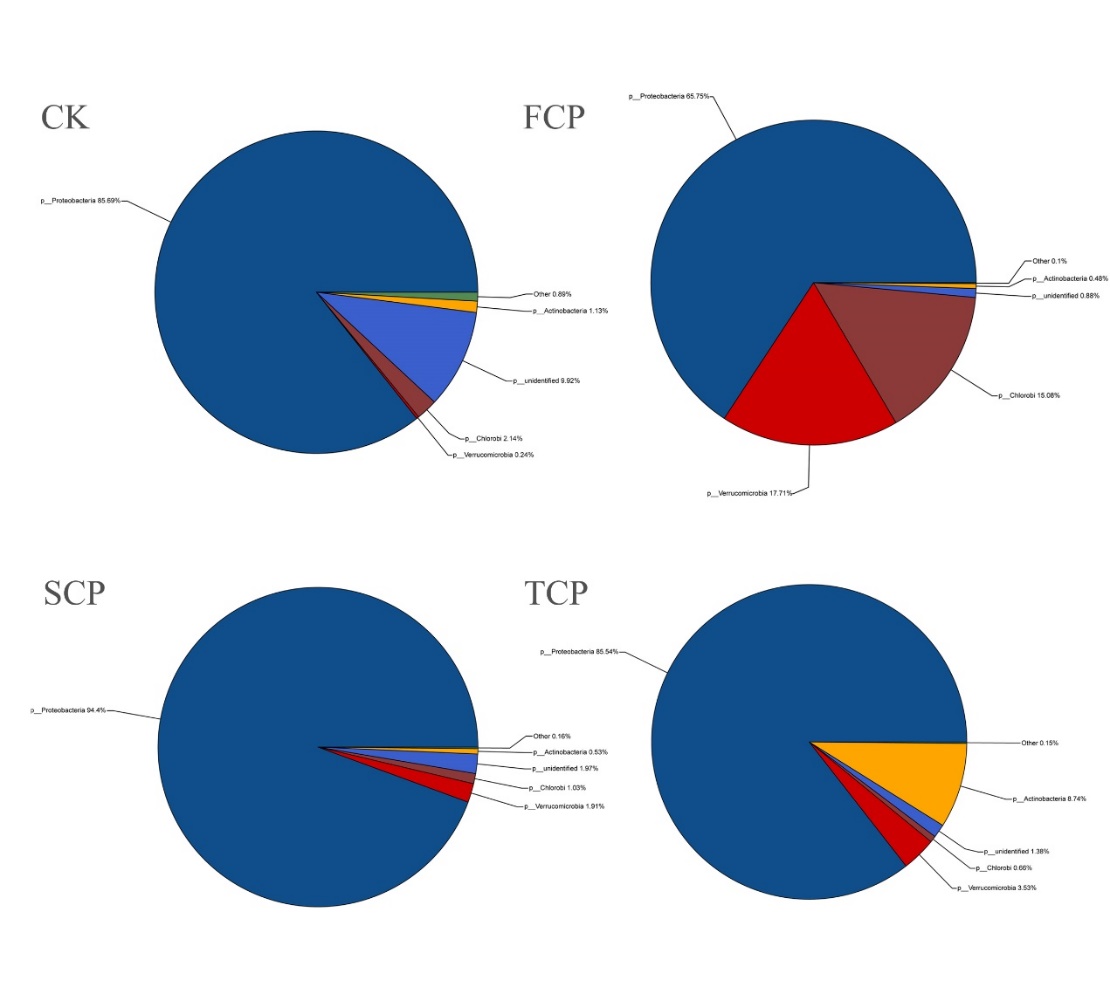


**Figure S4.** Pie chart of predominant nitrogen-fixing microbial communities (at the phylum level) in four individual soil samples.

**Table S1.** The soil physicochemical properties of different rotation plantations

| Categories | pH | TN  (mg·kg−1) | AN (mg·kg−1) | TP (mg·kg−1) | AP (mg·kg−1) | TK  (mg·kg−1) | AK (mg·kg−1) |
| --- | --- | --- | --- | --- | --- | --- | --- |
| CK | 5.18±0.01a | 433.65±19.63b | 65.00±2.00b | 370.34±7.59b | 1.22±0.02a | 2012.29±14.49a | 50.12±0.25a |
| FCP | 5.15±0.01b | 515.98±32.38a | 73.33±2.08a | 390.05±6.66a | 1.35±0.05a | 2125.95±228.27a | 49.30±5.03a |
| SCP | 4.92±0.01d | 436.80±30.58b | 68.33±7.09ab | 392.28±1.39a | 1.23±0.03a | 2041.81±63.11a | 50.43±5.00a |
| TCP | 4.95±0.01c | 469.03±31.85ab | 66.00±1.73ab | 374.06±9.35b | 1.34±0.15a | 2052.33±48.20a | 50.66±0.47a |

Note: the different letters in each column represent significant differences (*P* ≤ 0.05, n = 3).

**Table S2.** Information regarding tags and OTUs clusters in 12 different soil samples.

| Treatments | Raw tags | Clean tags | OTUs |
| --- | --- | --- | --- |
| CK1 | 76255 | 59580 | 270 |
| CK2 | 69739 | 55445 | 286 |
| CK3 | 56059 | 47546 | 259 |
| FCP1 | 81110 | 75508 | 384 |
| FCP2 | 101498 | 96352 | 333 |
| FCP3 | 77002 | 73734 | 278 |
| SCP1 | 28803 | 25887 | 356 |
| SCP2 | 45209 | 42191 | 356 |
| SCP3 | 35443 | 33453 | 412 |
| TCP1 | 30036 | 28975 | 570 |
| TCP2 | 36701 | 35559 | 530 |
| TCP3 | 85825 | 82369 | 576 |

Note: CK, FCP, SCP and TCP represent the control with no *C. equisetifolia* cultivation, the first rotation plantation, the second rotation plantation and the third rotation plantation. Different letters in each column indicate significant differences (*P* ≤ 0.05, n=3).
